# Multi-omics integration identifies ASPH and PTTG1 as potential causal drivers of lung adenocarcinoma progression and immune evasion

**DOI:** 10.3389/fimmu.2025.1689275

**Published:** 2025-11-12

**Authors:** Kai Yang, MeiFeng Chen, Yao Wu, WenJuan Duan, Na Huang, Xia Zhao, DeYun Cheng

**Affiliations:** 1 Department of Respiratory and Critical Care Medicine, West China Hospital, Sichuan University, Chengdu, Sichuan, China; 2 Department of Respiratory and Critical Care Medicine, First Affiliated Hospital of Chengdu Medical College, Chengdu, Sichuan, China; 3 Sichuan Provincial Clinical Medical Research Center for Radiology and Therapy, Chengdu, Sichuan, China; 4 Key Laboratory of Geriatric Respiratory Diseases of Sichuan Higher Education Institutes, Chengdu, Sichuan, China; 5 Department of Orthopedics, The First Affiliated Hospital of Chengdu Medical College, Chengdu, Sichuan, China

**Keywords:** lung adenocarcinoma (LUAD), ASPH, PTTG1, angiogenesis, single-cell RNA-seq, Mendelian randomization, prognostic model

## Abstract

**Background:**

Despite advances in therapy, lung adenocarcinoma (LUAD) remains a leading cause of cancer mortality. Angiogenesis and immune evasion critically influence LUAD progression and treatment resistance, yet epithelial-derived regulatory mechanisms and causal genes remain unclear.

**Methods:**

We employed single-cell transcriptomics (scRNA-seq) to identify angiogenesis-related epithelial-specific genes in LUAD. Mendelian randomization (MR) analyses utilizing large-scale genomic databases (eQTLGen, FinnGen) established genetic causality. A prognostic risk model was developed and validated using GEO and TCGA cohorts. Western blotting in clinical specimens and functional assays (gene knockdown, proliferation, migration, and invasion) verified core gene functions.

**Results:**

Aspartate β-hydroxylase (ASPH) and Pituitary tumor-transforming gene 1 (PTTG1) were identified as causal genes linked to LUAD risk and poor prognosis. Elevated protein expression of ASPH and PTTG1 was confirmed in LUAD tissues. ASPH knockdown significantly inhibited LUAD cell proliferation, migration, and invasion. The ASPH/PTTG1-based risk model robustly predicted prognosis. High-risk patients demonstrated “cold” immune microenvironments characterized by increased stromal infiltration and reduced immune effector cells. These patients also showed heightened sensitivity to several chemotherapeutic and targeted agents, including Cisplatin and Crizotinib.

**Conclusion:**

Integrating single-cell sequencing, MR-based causality, clinical validation, and functional experiments, we identified ASPH and PTTG1 as key regulators of LUAD angiogenesis and immune evasion. These findings substantiate ASPH/PTTG1 as promising biomarkers and therapeutic targets, offering new insights into precision therapies integrating anti-angiogenic and immunomodulatory strategies.

## 1 Introduction

Recent epidemiological data on cancer worldwide indicates that lung cancer impacts approximately two million people annually, with close to half of these cases occurring within Asian demographics (1). Among the different histological types, LUAD stands out as the most prevalent subtype, accounting for nearly 40% of all lung cancer diagnoses (2). Despite the progress made in diagnostic methods, targeted treatments, and immunotherapy, individuals diagnosed with LUAD still encounter a poor overall prognosis. As reported by the American Cancer Society in 2023, the five-year survival rate for lung cancer is only 23.6% (3). Therefore, elucidating the key pathogenic mechanisms underlying LUAD and developing precise and reliable prognostic biomarkers and risk assessment models are critical for accurately predicting disease progression, guiding individualized treatment strategies, and playing a significant role in improving survival rates as well as enhancing the overall quality of life for individuals affected by the condition.

Angiogenesis is defined as the development of new capillary networks originating from existing blood vessels. This process is essential for tumor initiation, aggressive growth, and distant metastasis, and represents a hallmark of advanced-stage cancers (4). Hypoxia within the tumor microenvironment is one of the major driving forces of angiogenesis. It stabilizes hypoxia-inducible factors (such as HIF-1α and HIF-2α), consequently resulting in the augmented expression of essential genes that play a crucial role in angiogenesis, such as VEGF and its receptors (VEGFR), neuropilin (NRP), epidermal growth factor (EGF), and angiopoietins (ANG), among others (5). Among these, VEGF is considered a central regulator of angiogenesis, as it not only significantly increases vascular permeability within tumors but also directly promotes neovascularization (6).

In addition to its angiogenic functions, VEGF is also acknowledged for its crucial role in regulating immune interactions within the tumor microenvironment (TME) (7, 8). While VEGF is primarily secreted by vascular endothelial cells, various immune cells in the TME—including tumor-associated macrophages and neutrophils (9), natural killer (NK) cells and mast cells (MCs)-also contribute to VEGF production (10–14). In non-small cell lung cancer, tumor-associated macrophages (TAMs) predominantly exhibit an M2 polarization. This specific phenotypic orientation promotes tumor proliferation and metastasis by increasing the secretion of VEGF (7). Concurrently, VEGF secreted by lung cancer cells themselves can activate several pro-proliferative signaling pathways, such as the MEK/ERK and PI3K/AKT pathways, further enhancing tumor cell invasion and growth (15).

Over the past two decades, anti-angiogenic drugs (AADs) targeting VEGF-A have been widely applied in therapeutic strategies targeting multiple tumor types (16, 17). However, due to the complex mechanisms of tumor angiogenesis and the high heterogeneity of the TME, current AADs face significant challenges, including limited applicability and the development of resistance (18–20). Therefore, elucidating the regulatory mechanisms of tumor angiogenesis and identifying novel key modulators and biomarkers are of great clinical significance for optimizing anti-angiogenic strategies and overcoming current therapeutic limitations.

LUAD is a malignant epithelial tumor in which epithelial cells secrete various growth factors, cytokines, and chemokines that directly or indirectly regulate essential biological processes in the TME, including angiogenesis. Although a number of studies have explored the relationship between LUAD and angiogenesis, few have systematically focused on epithelial cell–specific angiogenic regulators in LUAD, particularly from a multidimensional perspective integrating causality and clinical relevance. To address this gap, the present study employs an integrative approach combining scRNA-seq, MR, and bulk RNA sequencing to identify and validate key epithelial cell–derived angiogenic regulators in LUAD and to construct a prognostic evaluation model. This multi-omics strategy not only enhances the robustness and biological relevance of the findings but also facilitates the identification of clinically meaningful biomarkers, providing a strong foundation for future therapeutic and prognostic applications.

## 2 Materials and methods

### 2.1 Data acquisition and preprocessing

Transcriptomic data (TPM format) and corresponding clinical information for LUAD were downloaded from TCGA using the R package TCGAbiolinks (v2.26.0), and served as the training cohort. This dataset included 59 adjacent normal tissue samples and 541 tumor samples. Meanwhile, the Gene Expression Omnibus (GEO) datasets GSE37745 (n=106) and GSE41271 (n=182) were obtained as validation cohorts. Preprocessing steps included: (1) excluding samples with missing or zero survival information; (2) removing genes with >50% missing expression values; and (3) filtering out genes not expressed in more than 50% of the samples.

### 2.2 eQTL data collection and filtering

Cis-expression quantitative trait loci (cis-eQTL) datasets derived from whole blood were sourced from the publicly accessible eQTLGen Consortium resource. For the purpose of conducting MR analysis, instrumental variables were determined by choosing single nucleotide polymorphisms that satisfied the genome-wide significance criterion (p < 5 × 10^-8^) and underwent linkage disequilibrium filtering, with a clumping distance set at 10,000 kb and an r² threshold of less than 0.1.

### 2.3 GWAS summary statistics for LUAD outcomes

Summary statistics pertaining to a genome-wide association study (GWAS) focused on LUAD were obtained from the FinnGen Consortium (Release 12, https://www.finngen.fi/en). The dataset comprises 2,219 individuals diagnosed with LUAD alongside a control group consisting of 378,749 individuals of European ancestry.

### 2.4 scRNA-seq data processing

The scRNA-seq dataset GSE131907, which includes paired LUAD tumor and adjacent normal tissues from 11 patients, was downloaded from the GEO database. Data processing was performed using the Seurat R package (v5.1.0) following standard workflows, including log-normalization, identification of highly variable genes, dimensionality reduction via principal component analysis (PCA), and cell clustering. Cell type annotation was subsequently performed using the SingleR package (v2.4.1).

### 2.5 Angiogenesis gene set activity (AUC) analysis

A total of 5,928 angiogenesis related genes were compiled from the GeneCards database. The AUCell R package (v1.24.0) was used to calculate the AUC score for each cell based on this gene set. AUC values were visualized on the t-SNE plot to assess angiogenesis activity at the single-cell level.

### 2.6 Cell–cell communication analysis

The interaction dynamics between epithelial cells and various cellular elements within their microenvironment. Ligand–receptor interaction strength was computed and visualized using heatmaps. The relative strength of both outgoing and ingoing signaling pathways across different cell populations was assessed.

### 2.7 Pseudotime trajectory analysis

Pseudotime analysis was conducted using the Monocle2 (v2.30.1) R package, focusing on genes with high expression and dispersion. Cellular trajectory mapping was performed to infer dynamic transitions, particularly within epithelial subpopulations.

### 2.8 Differential expression analysis

In order to discern differentially expressed genes (DEGs) between LUAD tumor specimens and their corresponding adjacent normal tissues in the TCGA dataset, we utilized the DESeq2 R package (version 1.42.1). A gene was classified as significantly dysregulated if it demonstrated an absolute log_2_ fold change of at least 1, accompanied by a false discovery rate (FDR) of less than 0.05.

### 2.9 Key gene selection

Venn diagram analysis was conducted to identify overlapping genes among three gene sets: (1) TCGA-derived DEGs; (2) epithelial cell–specific marker genes from the scRNA-seq analysis; and (3) angiogenesis-related genes from GeneCards. Genes in the intersection were defined as “angiogenesis-related DEGs specific to epithelial cells in LUAD.”

### 2.10 Two-sample mendelian randomization analysis

In order to explore the possible causal relationships between the candidate genes identified in Section 2.9 and the susceptibility to LUAD, a TSMR approach was utilized, employing the TwoSampleMR R package (version 0.6.10). Cis-expression quantitative trait loci (eQTLs) functioned as instrumental variables for gene expression (the exposure variable), while the outcome dataset comprised genome-wide association summary data for LUAD obtained from the FinnGen consortium. Variables deemed weakly influential, characterized by an F-statistic of less than 10, were excluded from the analysis. The primary analytical technique employed was the inverse variance weighted (IVW) method. To assess horizontal pleiotropy, the MR-Egger intercept test was conducted, whereas Cochran’s Q statistic was used to evaluate heterogeneity. Additionally, a leave-one-out (LOO) analysis was carried out to verify the robustness of the causal estimates.

### 2.11 Construction and validation of the prognostic risk model

In the TCGA training dataset, both multivariate Cox and univariate Cox proportional hazards regression analyses were performed to identify genes that exhibit significant correlations with overall survival (OS), drawing from the candidates delineated in Section 2.9. Subsequently, a prognostic risk score model was formulated utilizing the following equation: Risk Score=β_1_ ×X_1_ +β_2_×X_2_ +…+β_n_ ×X_n_ , where β signifies the regression coefficient and X indicates the expression level of the respective gene.

Using the median risk score, patients diagnosed with LUAD were categorized into high and low risk subgroups. To evaluate the prognostic relevance of this model, Kaplan–Meier survival analysis was conducted within the TCGA dataset, as well as in two external GEO validation cohorts (GSE37745 and GSE41271), utilizing the survival R packages (version 0.4.9).

### 2.12 Association between clinical features and Risk Score

Associations between the Risk Score and clinicopathological characteristics of TCGA_LUAD patients (e.g., age, TNM stage) were analyzed using t-tests, Wilcoxon rank-sum tests, or chi-square tests as appropriate.

### 2.13 Nomogram construction and evaluation

The independent prognostic factors determined through multivariate Cox regression—comprising both the risk score and various clinical parameters—were utilized to develop a nomogram aimed at forecasting OS at 1, 3, and 5 years. This was accomplished using the rms R package (version 67.1). The efficacy of the model and its clinical applicability were assessed by means of calibration plots, time-dependent receiver operating characteristic (ROC) curves, and decision curve analysis (DCA).

### 2.14 Tumor immune microenvironment analysis

The variations in immune infiltration between groups categorized as high and low-risk were analyzed using the CIBERSORT and ssGSEA algorithms, which were executed through the GSVA R package (version 1.50.5). In addition, the ImmuneScore, StromalScore, and ESTIMATEScore were utilized the ESTIMATE R package (version 1.0.13) and were subsequently compared across different risk categories.

### 2.15 Gene set enrichment analysis

Gene set enrichment analysis (GSEA) was conducted utilizing the c2.all.v2024.1. Hs.symbols gene sets to investigate hallmark pathways that exhibit differential enrichment between high and low-risk cohorts. Pathways were considered statistically significant if they met the criteria of |normalized enrichment score (NES)| > 1 and p < 0.05.

### 2.16 Analysis of immunotherapy-related biomarkers

To evaluate the possible efficacy of immunotherapy, a comparative analysis was conducted on tumor mutational burden (TMB), cytolytic activity, as well as immune checkpoint genes between groups classified as high-risk and low-risk.

### 2.17 Drug sensitivity prediction

The oncoPredict R package was utilized to forecast drug sensitivity in samples from TCGA, leveraging information sourced from the Genomics of Drug Sensitivity in Cancer database (https://www.cancerrxgene.org/). The estimated half-maximal inhibitory concentration (IC_50_) values, as well as response scores for both chemotherapeutic and targeted therapies, were analyzed and compared between groups categorized as high-risk and low-risk.

### 2.18 Clinical sample collection and Western blot analysis

Specimens comprising six paired LUAD tumors alongside adjacent normal tissues were procured from pathologically verified patients at The First Affiliated Hospital of Chengdu Medical College. The Institutional Ethics Committee granted ethical approval for the study (Approval No.: 2025CYFYIRB-SQ-81), and all participants provided written informed consent.

Fresh-frozen tissue samples were subjected to pulverization in liquid nitrogen, followed by total protein extraction utilizing RIPA lysis buffer that was augmented with protease and phosphatase inhibitors. The quantification of protein concentrations was performed using a BCA assay kit (Thermo Fisher Scientific, USA). For analysis, equal quantities of protein (30 μg) were subjected to separation using 10% SDS-PAGE, and the proteins were subsequently transferred to PVDF membranes (Millipore, USA). The membranes were then blocked with a 5% non-fat milk solution in TBST at ambient temperature for one hour, before being incubated overnight at 4 °C with the designated primary antibodies: rabbit anti-ASPH (1:1000, 14116-1-AP, China), and rabbit anti-PTTG1 (1:1000, 18040-1-AP. After washing with TBST, membranes were incubated with HRP-conjugated goat anti-rabbit secondary antibody, the membranes were incubated for 1 hour at room temperature, and protein bands were visualized using enhanced chemiluminescence (ECL) reagents (Millipore, USA) and captured by a gel imaging system (Bio-Rad, USA). Semi-quantitative analysis was performed using ImageJ software.

### 2.19 Cell culture and gene knockdown

Human LUAD cell lines SW1573 and A549 were procured from the Cell Bank of the Chinese Academy of Sciences. These cell lines were selected as they are well-established and commonly used models for LUAD research, representing distinct molecular and phenotypic characteristics. These cell lines were supplemented with 10% fetal bovine serum, maintained in RPMI-1640 medium (Corning, CN), penicillin at a concentration of 100 U/mL, and streptomycin at 100 μg/mL. The cultures were incubated at 37 °C in a humidified environment with 5% CO_2_.

For the purpose of ASPH knockdown, lentiviral vectors that express short hairpin RNAs (shRNAs) targeting ASPH (specifically shASPH-1 and shASPH-2) alongside a negative control (shCtrl) were fabricated by GeneChem (Shanghai, China). The cells underwent transduction at an appropriate multiplicity of infection (MOI) for a duration of 48 hours, after which stable transfectants were selected using 2 μg/mL puromycin. The efficacy of the knockdown was subsequently verified through Western blot analysis.

### 2.20 Western blot analysis (cell protein detection)

Cells in the logarithmic growth phase were harvested, and total protein extraction was conducted utilizing RIPA lysis buffer that was enriched with protease and phosphatase inhibitors. Protein concentrations were determined employing a BCA assay kit, and equal quantities (30 μg) of protein were subjected to SDS-PAGE and subsequently transferred onto PVDF membranes (Millipore, USA). The membranes underwent blocking with 5% skim milk in TBST at ambient temperature for one hour, followed by an overnight incubation at 4°C with rabbit anti-ASPH and rabbit anti-GAPDH primary antibodies. Post-washing, the membranes were treated with an HRP-conjugated secondary antibody at room temperature for one hour. Signal detection was executed utilizing ECL reagents, and images were acquired with a gel documentation system. Band intensity quantification was carried out using ImageJ software.

### 2.21 CCK8 cell proliferation assay

Cell viability was determined using a CCK-8 assay. Briefly, stably transduced shCtrl and shASPH cells were seeded at 3,000 cells/well in 96-well plates. At specified time points (0, 24, 48, and 72 hours), 10 µL of CCK-8 solution was added to each well, followed by a 2-hour incubation. The absorbance at 450 nm was then measured using a microplate reader to assess cell viability.

### 2.22 Transwell invasion assay

Cellular invasion was assessed through the utilization of Transwell chambers coated with Matrigel (8 μm pore diameter, Corning, USA). Briefly, 5×10^4^ cells suspended in 200 μL serum-free medium were seeded into the upper chamber, with 20% FBS in the lower chamber acting as the chemoattractant. After 24 hours, non-invading cells were removed from the upper membrane, while invaded cells on the lower surface were fixed in 4% paraformaldehyde for 15 minutes before being stained with 0.1% crystal violet for 20 minutes. Invasion was quantified by averaging the cell count from five random microscopic fields.

### 2.23 Wound-healing assay

Cells were inoculated into 6-well plates and allowed to grow until they achieved 90-100% confluence. A linear scratch was created with a sterile 200 μL pipette tip, and subsequent washing with phosphate-buffered saline (PBS) was performed to eliminate any dislodged cells. The cells were then preserved in a serum free medium, and the process of wound healing was assessed by taking photographs at 0, 24, and 48 hours. Wound widths were measured using ImageJ, and migration rates (%) were calculated as follows: Migration rate (%)=[(initial wound width − wound width at time t)/initial wound width] × 100%.

### 2.24 Statistical analysis

All statistical analyses were performed using R software (version 4.3.3) and GraphPad Prism (version 9.0). For comparisons between two groups, Student’s t-test was used for normally distributed data, while the Wilcoxon rank-sum test was applied for non-normally distributed data. Associations between categorical variables were assessed using the chi-square test. Survival curves were generated using the Kaplan-Meier method and compared with the log-rank test. Univariate and multivariate Cox proportional hazards regression models were used to identify prognostic factors. A two-sided P-value < 0.05 was considered statistically significant. Significance levels in figures are denoted as follows: * P < 0.05, ** P < 0.01, and *** P < 0.001.

## 3 Results

### 3.1 Single-cell transcriptomics reveals the tumor microenvironment landscape of LUAD

#### 3.1.1 Cell subpopulation identification and annotation

**Figure 1:**
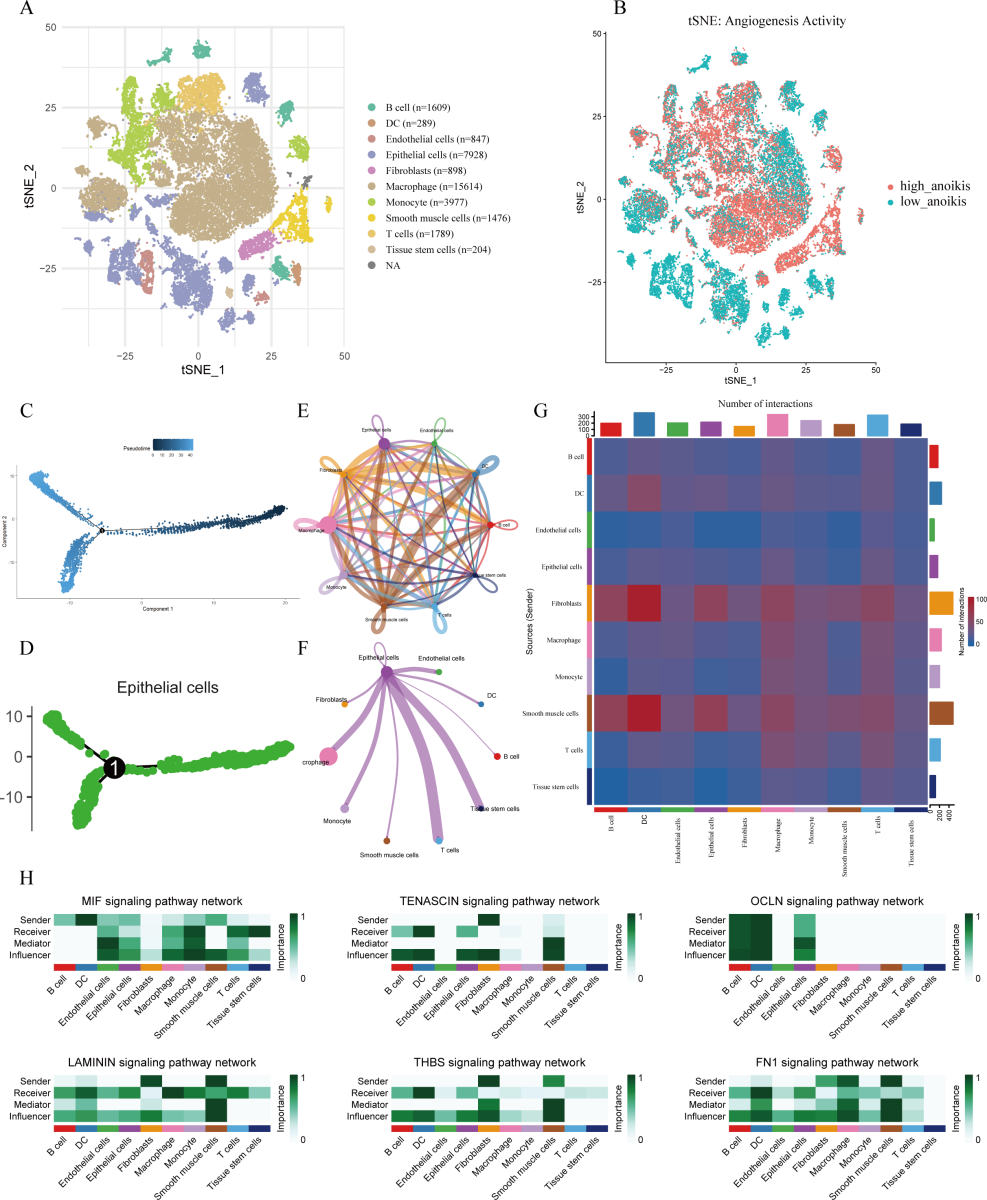
Single-cell transcriptomic analysis of LUAD. t-SNE visualization of cell clusters, annotated into 11 major cell types based on canonical marker genes. **(B)** Angiogenesis Activity Visualization. Visualization of angiogenesis-related AUC scores on the t-SNE plot; color indicates the relative score intensity. **(C)** Pseudotime trajectory analysis of all analyzed cell populations. **(D)** Pseudotime trajectory of epithelial subpopulations showing potential differentiation paths. **(E)** Global overview of intercellular communication networks among all cell types. **(F)** Cell-cell interaction network centered on epithelial cells. **(G)** Heatmap showing interaction intensity among cell types. **(H)** Outgoing and incoming signaling patterns of key pathways across cell types, with emphasis on epithelial cells.

To investigate the cellular heterogeneity of LUAD, we performed scRNA-seq analysis on the GSE131907 dataset. Using t-SNE for dimensionality reduction and visualization, cells were clearly clustered into 11 distinct groups. Cell populations were classified into 11 primary types, which encompass fibroblasts, epithelial cells, and macrophages, utilizing the expression patterns of well-characterized marker genes specific to each cell type (**Figure 1A**). The activity of the angiogenesis-related gene set was assessed using the AUCell algorithm, revealing relatively high enrichment scores in macrophages, fibroblasts, smooth muscle cells, and epithelial cells (**Figure 1B**). We further constructed pseudotime trajectories to explore gene expression dynamics during LUAD progression (**Figure 1C**). Specifically, a pseudotime trajectory was successfully reconstructed for epithelial cell subpopulations (**Figure 1D**).

#### 3.1.2 Cell–cell communication network analysis

Intercellular communication networks were inferred using the CellChat package. Significant signaling interactions were observed among epithelial cells, macrophages, T cells, and tissue stem cells, with these populations acting as both major signal senders and receivers (**Figures 1E, G**). Notably, epithelial cells served as a central communication hub within the network (**Figure 1F**). Analysis of incoming and outgoing signaling patterns revealed that epithelial cells were key recipients and transmitters of multiple signaling pathways—including MIF, TENASCIN, OCLN, LAMININ, THBS, and FN1—all of which are known to play roles in angiogenesis regulation (**Figure 1H**).

### 3.2 Identification of angiogenesis-related differentially expressed genes in LUAD epithelial cells

**Figure 2:**
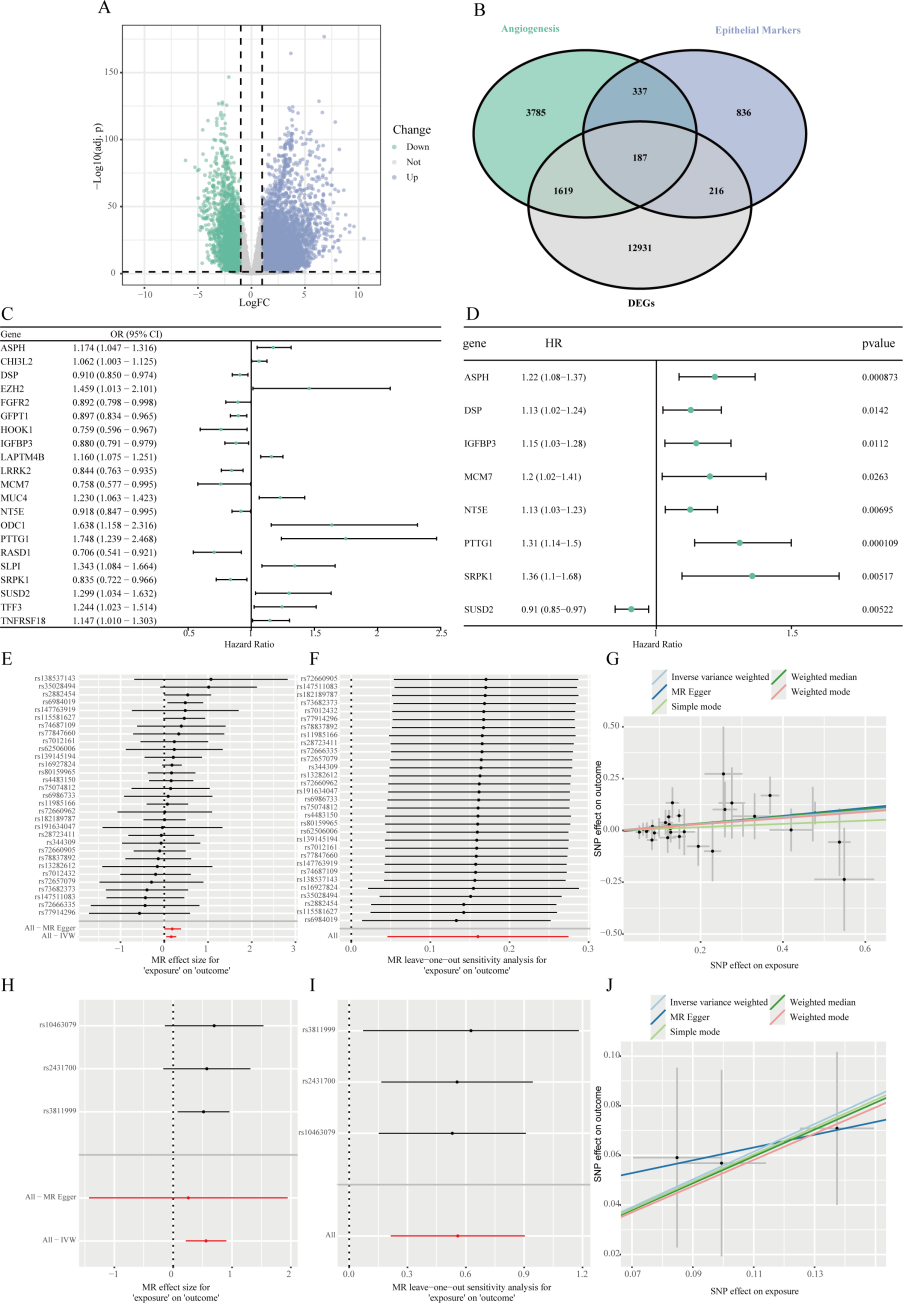
Identification of angiogenesis-related hub genes in LUAD epithelial cells and MR analysis. **(A)** A volcano plot demonstrating the genes that are DEGs when comparing lung adenocarcinoma tissues to corresponding adjacent normal samples within the TCGA-LUAD dataset. **(B)** A Venn diagram illustrating the intersection of DEGs identified from the TCGA dataset, epithelial cell-specific marker genes, and genes linked to angiogenesis. **(C)** Forest plot of significantly associated genes from MR analysis among intersecting genes with cis-eQTL data, evaluating the causal relationship between their expression and LUAD risk. **(D)** Forest plot of positively associated genes identified by univariate Cox regression analysis of MR-significant genes, assessing their correlation with OS. **(E-G)** Equivalent plots for ASPH, including forest plot **(E)**, leave-one-out analysis **(F)**, and MR scatter plot **(G)**. **(H-J)** Equivalent plots for PTTG1, including forest plot **(H)**, leave-one-out analysis **(I)**, and MR scatter plot **(J)**.

Differential expression analysis between LUAD normal and tumor tissues in the TCGA cohort identified 14,953 DEGs, with 3,296 downregulated and 11,657 upregulated in tumors (**Figure 2A**). By integrating 1,576 epithelial cell marker genes identified from scRNA-seq analysis and 5,928 angiogenesis-related genes from the GeneCards database, a Venn diagram was used to identify 187 overlapping genes that were differentially expressed, epithelial cell–specific, and angiogenesis-related (**Figure 2B**).

### 3.3 MR and identification of hub genes

Among the 187 intersecting genes, 118 had available cis-eQTL data in the eQTLGen database. TSMR analysis was conducted to evaluate the causal relationship between gene expression and LUAD risk. This analysis identified 21 genes whose genetically predicted expression levels were significantly associated with LUAD risk (P < 0.05). Of these, higher expression of 10 genes was associated with increased LUAD risk, while higher expression of 11 genes was associated with decreased risk (**Figure 2C**).

To further refine these candidates, we first performed a univariate Cox regression analysis on these 21 genes, which identified 8 genes significantly associated with OS (P < 0.05) (**Figure 2D**). From this subset, we selected genes where the direction of effect was consistent between the MR and survival analyses. Specifically, we required genes identified as risk factors in MR (OR > 1) to also be associated with poorer survival (HR > 1), and protective factors (OR < 1) with better survival (HR < 1). This stringent criterion ultimately pinpointed Aspartate β-hydroxylase (ASPH) and Pituitary tumor-transforming gene 1 (PTTG1) as the core hub genes. The primary IVW results indicated that genetically predicted higher expression of ASPH (OR=1.174, 95% CI: 1.047–1.316, P < 0.05) and PTTG1 (OR=1.748, 95% CI: 1.239–2.468, P < 0.05) was significantly associated with an increased risk of LUAD.

Primary MR results showed that genetically predicted higher expression levels of ASPH and PTTG1 were significantly associated with increased LUAD risk (**Figures 2E, H**). Leave-one-out sensitivity analysis showed that the estimated causal effect of ASPH and PTTG1 on LUAD risk remained stable when omitting any single SNP, confirming the robustness of the results (**Figures 2F, I**). Results from multiple MR methods, including weighted median and IVW, were consistent. MR-Egger regression didn’t detect significant horizontal pleiotropy (intercept P > 0.05) (**Figures 2G, J**).

### 3.4 Validation of hub gene expression

**Figure 3:**
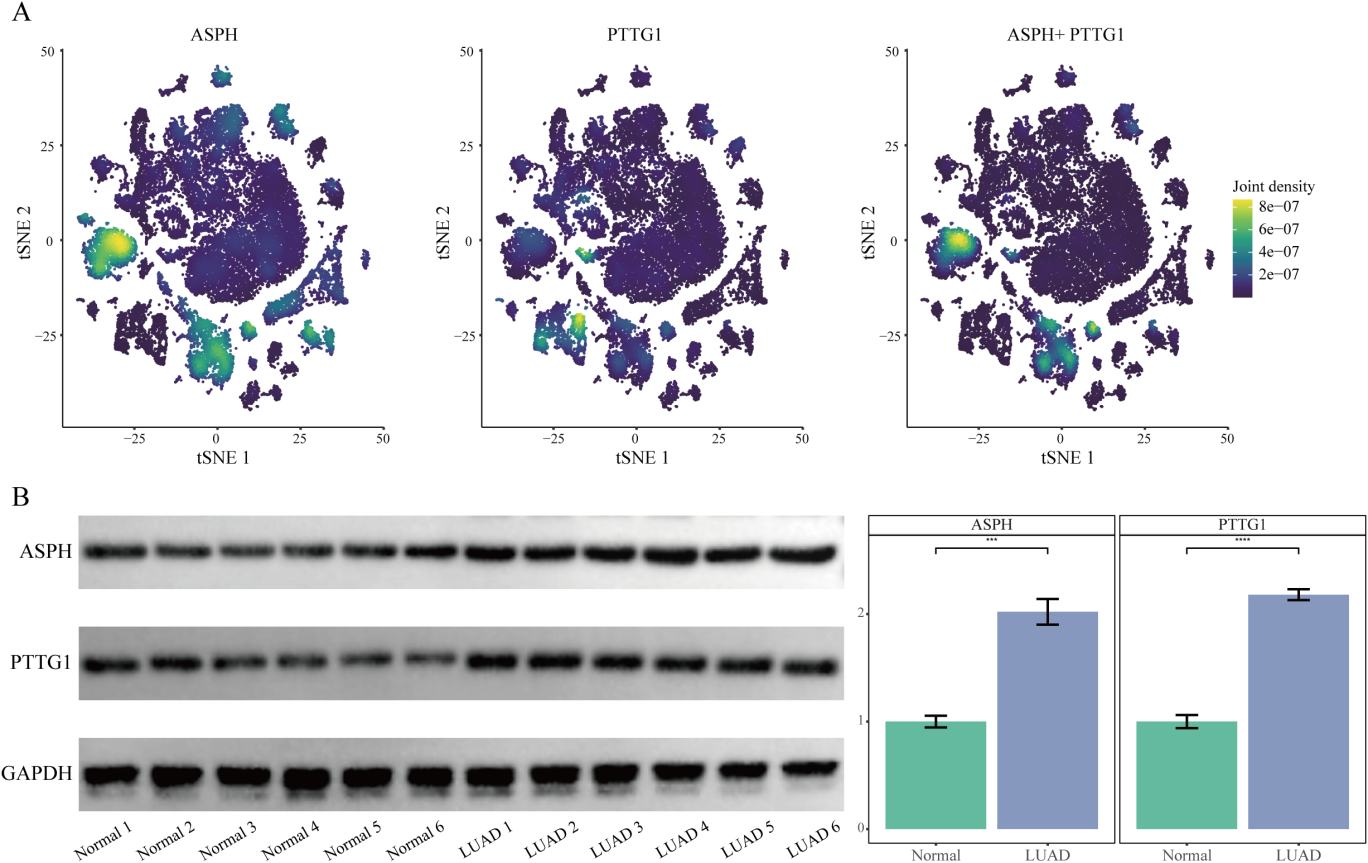
Validation of ASPH and PTTG1 expression. **(A)** t-SNE plots overlaid with expression densities of ASPH, PTTG1, and their combined signature. Color intensity reflects expression levels. **(B)** Western blot analysis of ASPH and PTTG1 protein expression in Lung Adenocarcinoma Tissue and Normal Adjacent Tissue.

In the single-cell t-SNE plot, ASPH and PTTG1 expression was predominantly enriched in epithelial cell and macrophage subpopulations (**Figure 3A**). Western blot analysis revealed that the protein expression levels of ASPH and PTTG1 were significantly higher in tumor tissues compared to adjacent normal tissues across all six paired LUAD samples (P < 0.001) (**Figure 3B**). This consistent upregulation observed in all cases further supports the potential clinical value of ASPH and PTTG1 as important molecular markers in LUAD.

### 3.5 Knockdown of ASPH inhibits malignant phenotypes of LUAD cells *in vitro*

**Figure 4:**
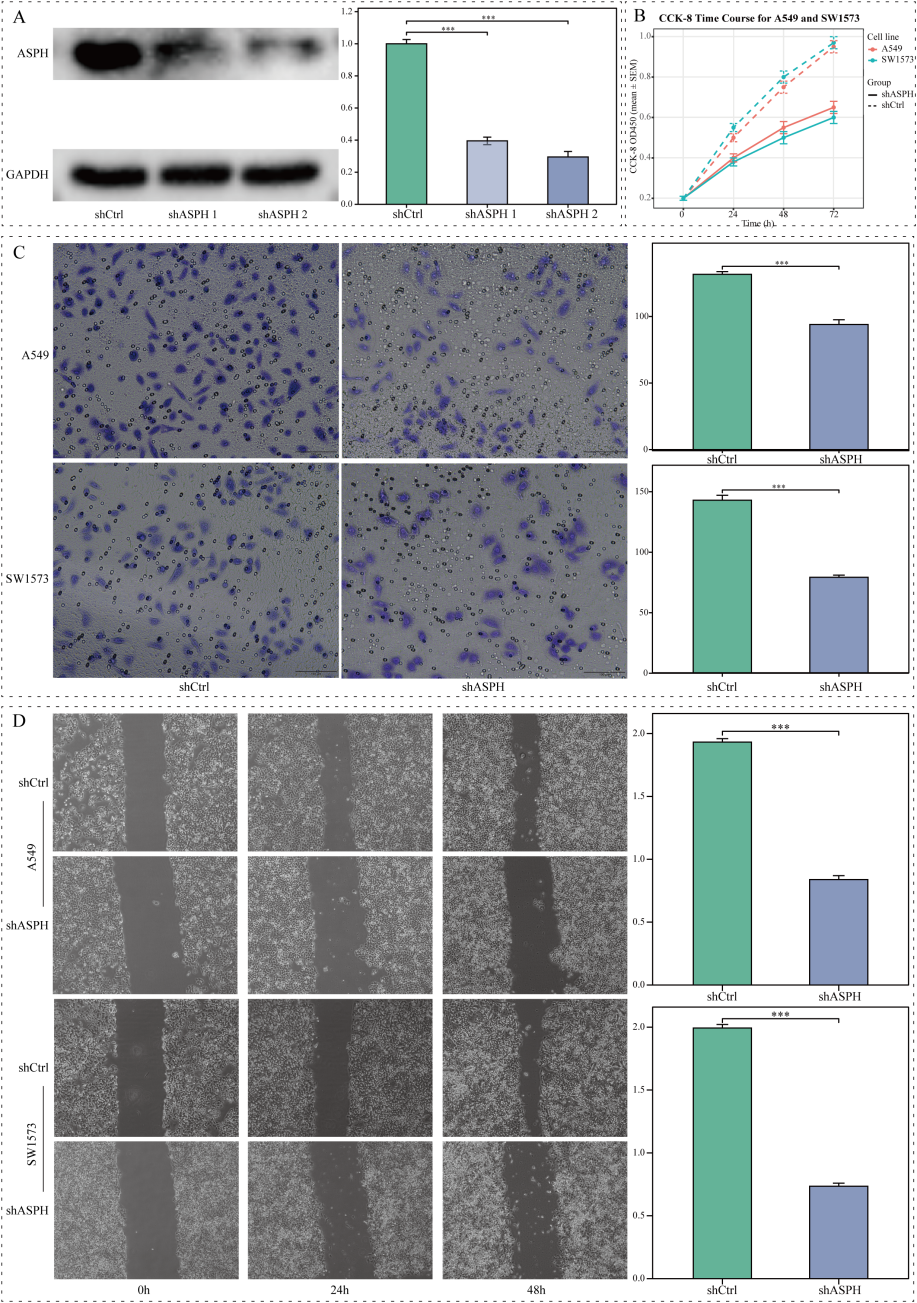
The impact of ASPH knockdown on the biological behavior of lung adenocarcinoma cells *in vitro*. **(A)** Western blot and corresponding grayscale analysis demonstrating ASPH expression levels in A549 cells that were transfected with shCtrl, shASPH-1, or shASPH-2. **(B)** CCK8 assay assessing the proliferation rates of A549 and SW1573 cells post-ASPH knockdown. **(C)** Transwell assay results indicating a reduction in invasion capabilities of ASPH-silenced A549 and SW1573 cells (scale bar=100 μm). **(D)** Wound-healing assay results revealing impaired migratory abilities following ASPH knockdown (scale bar=200 μm). Data are expressed as mean ± SD. ** P < 0.01, *** P < 0.001.

To directly validate the functional role of ASPH in LUAD cells, we knocked down its expression in SW1573 and A549 cell lines using lentivirus-mediated shRNA. Western Blot results confirmed that, compared to the shCtrl control group, two independent shRNAs (shASPH-1 and shASPH-2) effectively reduced ASPH protein levels. The more efficient shASPH-1 was selected for subsequent functional experiments (**Figure 4A**). The CCK8 proliferation assay, visualized as a line graph, revealed that ASPH knockdown led to a significant, time-dependent inhibition of proliferative capacity in both A549 and SW1573 cells compared to the control group (**Figure 4B**). Subsequently, we assessed the impact of ASPH on cell invasion and migration using wound-healing and Transwell assays. The results showed that ASPH knockdown significantly reduced the number of cells that invaded through the Matrigel matrix (**Figure 4C**) and markedly delayed the closure of the scratch area (**Figure 4D**). Collectively, these *in vitro* results confirm that ASPH is a critical molecule required for maintaining the proliferation, migration, and invasion of LUAD cells, providing strong functional support for our bioinformatics analyses and clinical association findings.

### 3.6 Validation and development of a prognostic risk model utilizing hub genes

**Figure 5:**
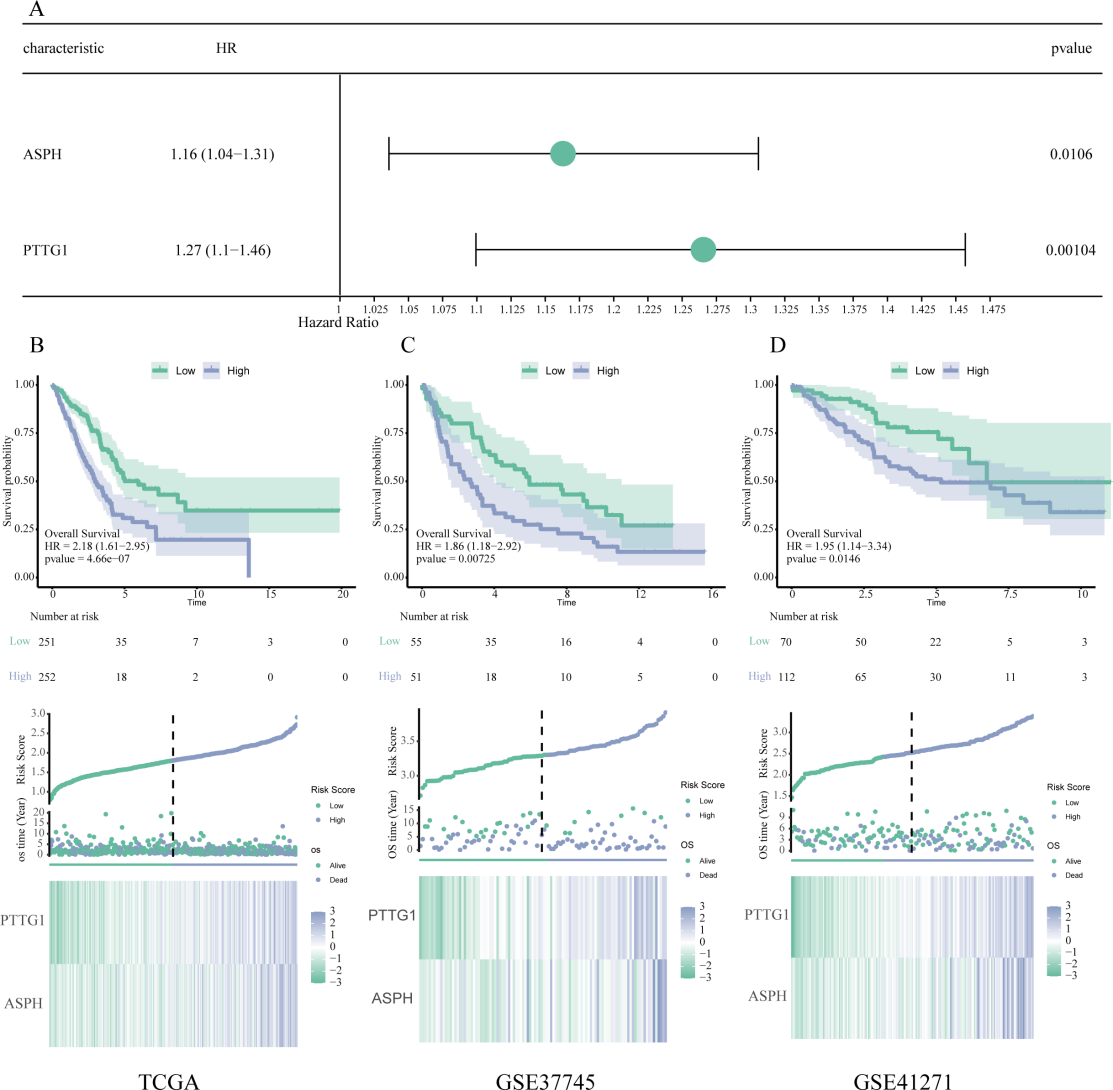
Validation and development of a prognostic model based on key genes. **(A)** Forest plot generated from multivariate Cox regression analysis, showcasing the hazard ratios and 95% confidence intervals for ASPH and PTTG1, thereby affirming their roles as independent prognostic indicators. **(B–D)** Assessment of the prognostic model using data from the TCGA **(B)**, GSE37745 **(C)**, and GSE41271 **(D)** datasets. Each subsection contains a Kaplan–Meier survival analysis (top), distribution plots of risk scores and survival outcomes (middle), as well as expression heatmaps for ASPH and PTTG1 (bottom).

A prognostic risk scoring model was developed that incorporated ASPH and PTTG1, which were determined through multivariate Cox analysis within the TCGA cohort (refer to **Figure 5A**). The formula for the model is defined as follows: Risk Score=(0.151 × Expression level of ASPH)+(0.236 × Expression level of PTTG1). Patients were categorized into high and low risk groups based on the median risk score. Kaplan–Meier survival analysis indicated a significantly diminished OS in the high-risk cohort when compared to the low-risk cohort, observed in both the TCGA training dataset (illustrated in **Figure 5B**) and two external GEO validation cohorts, namely GSE37745 (depicted in **Figure 5C**) and GSE41271 (shown in **Figure 5D**) (all P < 0.05). These findings underscore the robust prognostic significance and generalizability of the model.

### 3.7 Association between clinicopathological features and Risk Score

**Figure 6:**
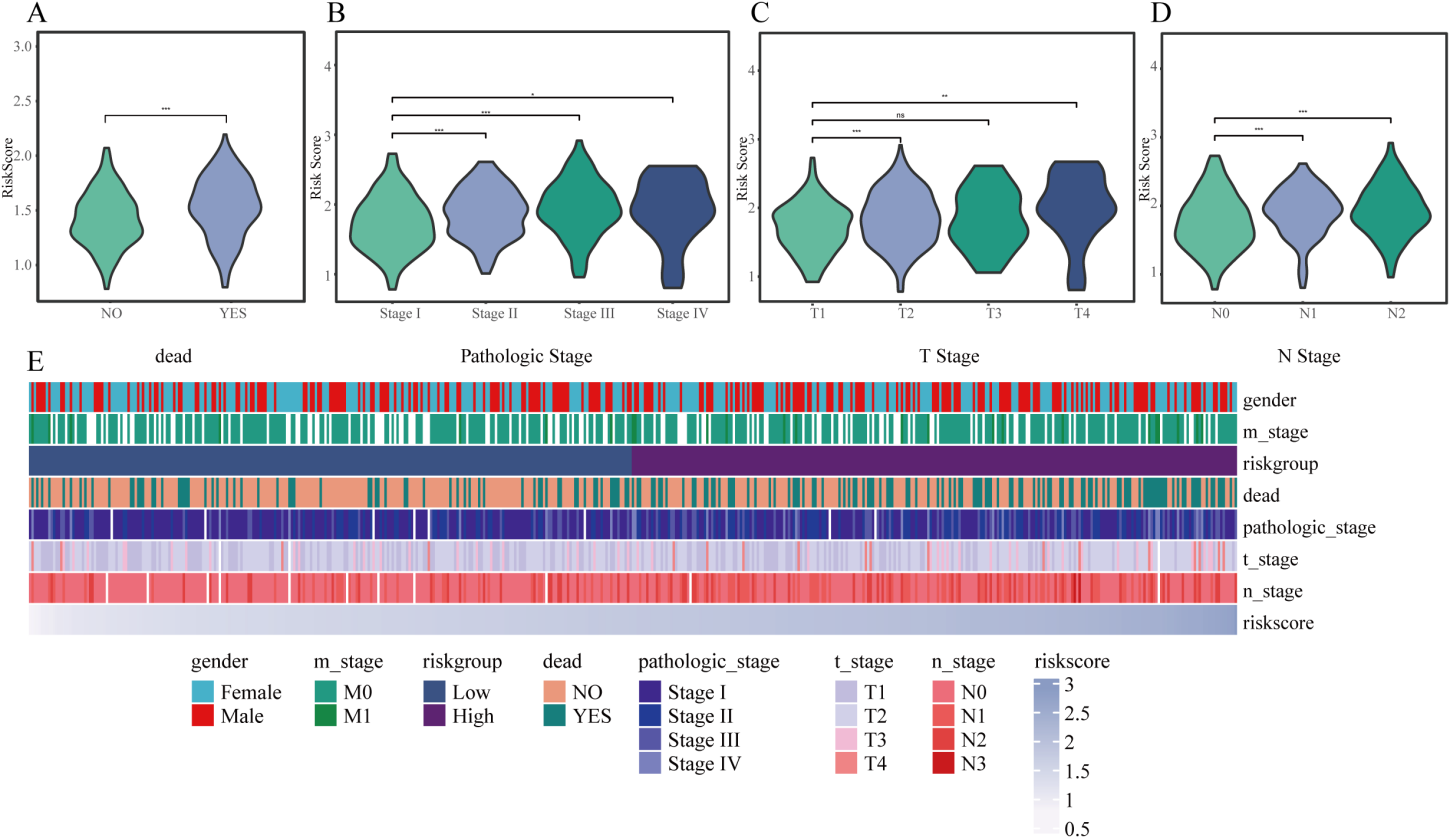
Correlation between risk score and clinical characteristics. **(A–D)** Violin plots illustrating variations in risk scores among different subgroups: **(A)** survival status, **(B)** pathological stage, **(C)** T classification, and **(D)** N classification. **(E)** Heatmap depicting the relationships between risk score and clinical variables such as sex, stage, and prognosis. * P < 0.05, ** P < 0.01, *** P < 0.001.

In the TCGA cohort, associations between the clinical features and risk score were analyzed. High risk scores were significantly associated with deceased status, advanced tumor stage, higher N stage, and T stage (all P < 0.05) (**Figures 6A–D**). These associations were further visualized using a heatmap integrating clinical features with the risk score (**Figure 6E**).

### 3.8 Assessment and development of a prognostic nomogram

**Figure 7:**
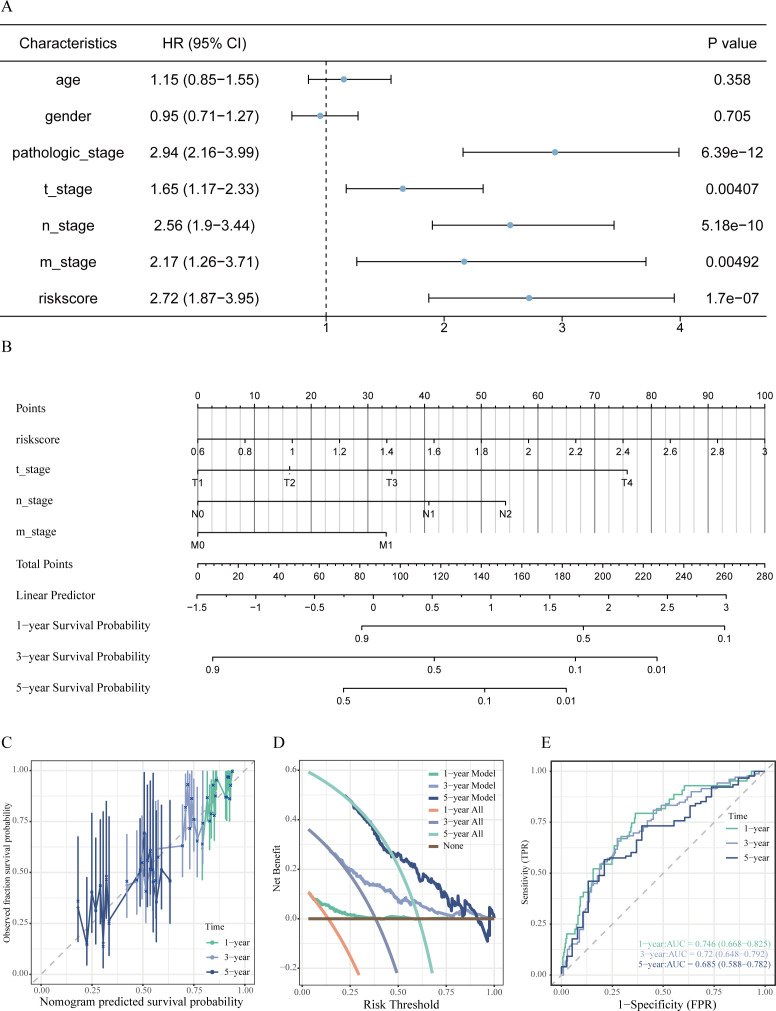
Development and assessment of the prognostic nomogram. **(A)** Forest plot from univariate Cox regression analysis identifying clinical factors linked to OS in LUAD patients. **(B)** Prognostic nomogram integrating risk score and significant clinical characteristics to predict 1-, 3-, and 5-year OS probabilities. **(C)** Calibration curves evaluating the alignment between predicted and actual OS at 1, 3, and 5 years. **(D)** Decision curve analysis assessing the net clinical benefit provided by the nomogram at various time points. **(E)** Time-dependent ROC curves along with associated AUC values illustrating the predictive efficacy of the model.

In order to create a customized prognostic instrument, a nomogram was developed that integrated the risk score along with various independent clinical prognostic factors identified through univariate Cox regression analysis (refer to **Figures 7A, B**). The nomogram displayed a high degree of concordance between the predicted survival outcomes and the actual survival data, as evidenced by the calibration curves (illustrated in **Figure 7C**). The decision curve analysis (DCA) indicated a significant clinical net benefit across a wide spectrum of threshold probabilities (depicted in **Figure 7D**). Furthermore, time-dependent ROC analysis indicated that the nomogram achieved area under the curve (AUC) values of 0.746, 0.720, and 0.685 for predicting OS at 1 years, 3 years, and 5 years, respectively (shown in **Figure 7E**).

### 3.9 Tumor immune microenvironment and Risk Score

**Figure 8:**
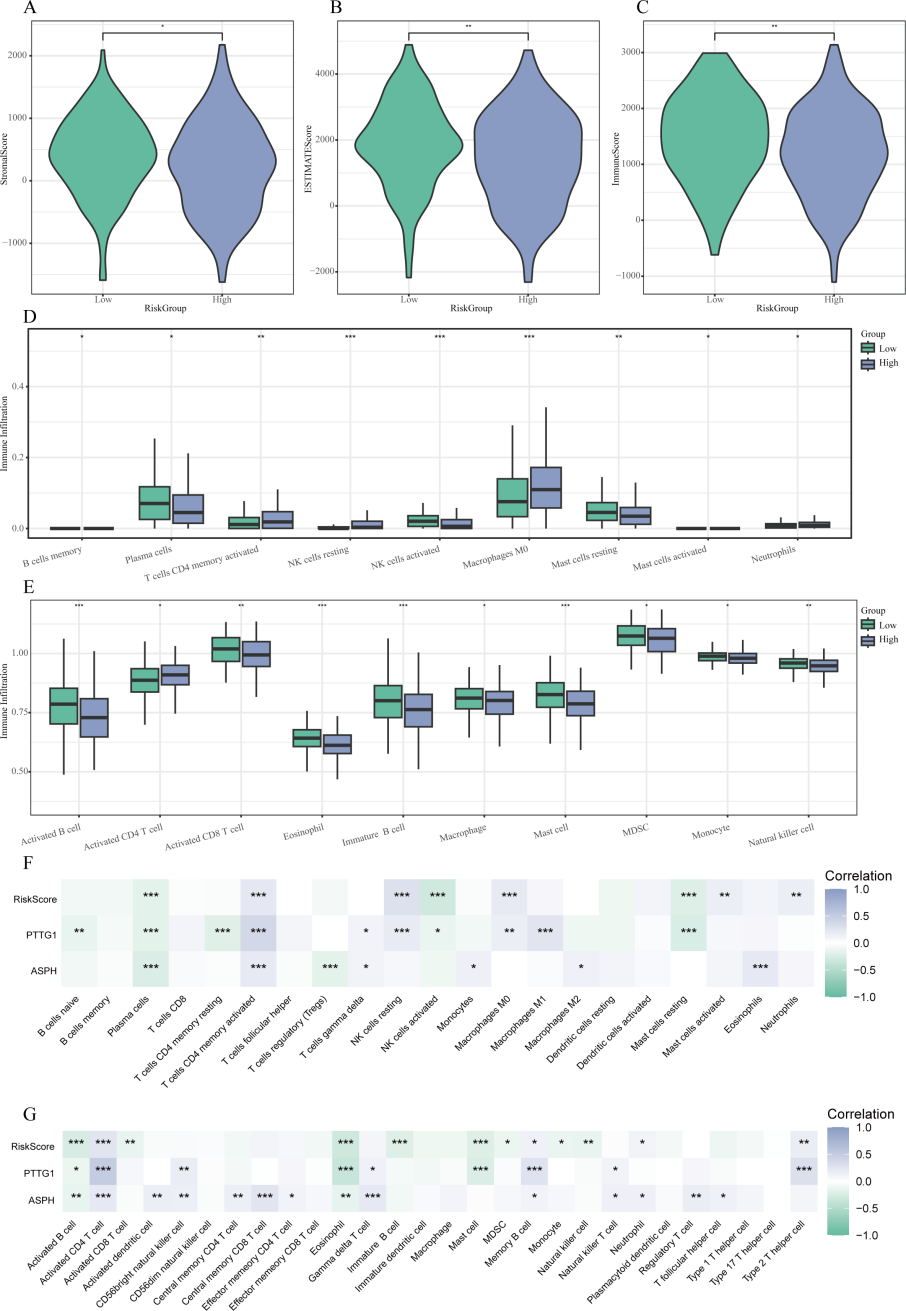
Relationship between characteristics and Risk Score of the tumor microenvironment. **(A-C)** Violin plots demonstrating variations in StromalScore **(A)**, ESTIMATEScore **(B)**, and ImmuneScore **(C)** among low- and high-risk cohorts. **(D)** Boxplots illustrating the patterns of immune cell infiltration assessed by ssGSEA across the different risk groups. **(E)** Boxplots presenting the disparities in immune cell infiltration as derived from CIBERSORT between the two groups. **(F-G)** Heatmaps that reveal correlations between the risk score, expression of hub genes, and immune cell populations evaluated through CIBERSORT **(F)** and ssGSEA **(G)**. * P < 0.05, ** P < 0.01, *** P < 0.001.

The implementation of the ESTIMATE algorithm indicated that patients classified as high-risk presented with markedly lower ImmuneScore and ESTIMATEScore, while exhibiting a heightened StromalScore (P < 0.05) (see **Figures 8A–C**). This suggests a tumor microenvironment that is rich in stroma but lacking in immune cell presence. Additional immune profiling conducted using CIBERSORT and single sample Gene Set Enrichment Analysis (ssGSEA) revealed a higher infiltration of activated memory CD4^+^ T cells, M0 macrophages, and resting natural killer (NK) cells within the high-risk cohort. In contrast, the levels of plasma cells, activated NK cells, resting mast cells, activated B cells, activated CD8^+^ T cells, and eosinophils were significantly reduced (all P < 0.05) (illustrated in **Figures 8D–G**).

### 3.10 Analysis of TMB, CYT scores, immune checkpoints, and GSEA

**Figure 9:**
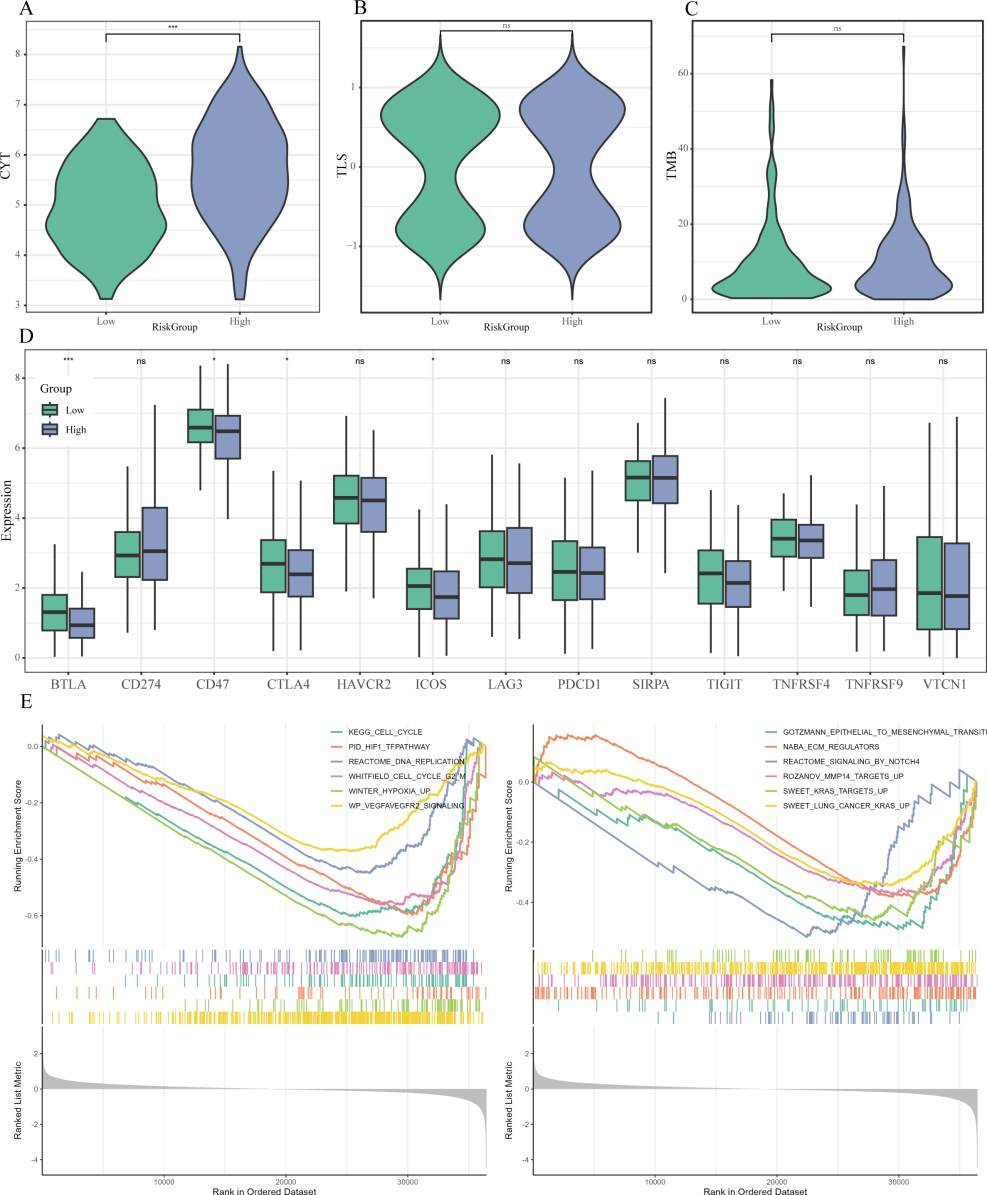
Immune therapy markers and pathway enrichment linked to Risk Score. **(A-C)** Violin plots examining the differences in cytolytic activity (CYT) scores **(A)**, tertiary lymphoid structure (TLS) scores **(B)**, and tumor mutational burden (TMB) **(C)** between risk categories. **(D)** Boxplots highlighting the expression variations of essential immune checkpoint molecules across the risk groups. **(E)** GSEA findings that display representative pathways that are significantly enriched in the high-risk category. * P < 0.05, ** P < 0.01, *** P < 0.001.

Subsequent analysis revealed that patients categorized as high-risk exhibited significantly elevated cytolytic activity (CYT) scores and tumor mutational burden (TMB) in comparison to their low-risk counterparts (P < 0.05; **Figures 9A–C**). Importantly, a notable decrease in the expression of several immune checkpoint genes, including BTLA, CD47, CTLA4, and ICOS, was observed within the high-risk cohort (P < 0.05) (**Figure 9D**).

Furthermore, the GSEA demonstrated that various hallmark pathways were notably enriched within the high-risk cohort, achieving a FDR of less than 0.05, including cell cycle regulation (e.g., KEGG_CELL_CYCLE), DNA replication (e.g., REACTOME_DNA_REPLICATION), hypoxia response (e.g., PID_HIF1_TFPATHWAY, WINTER_HYPOXIA_UP), angiogenesis (e.g., WP_VEGFAVEGFR2_SIGNALING), epithelial-mesenchymal transition (EMT) (e.g., GOTZMANN_EPITHELIAL_TO_MESENCHYMAL_TRANSITION), extracellular matrix remodeling (e.g., NABA_ECM_REGULATORS), and oncogenic KRAS signaling (e.g., SWEET_KRAS_TARGETS_UP) (**Figure 9E**).

### 3.11 Drug sensitivity prediction

**Figure 10:**
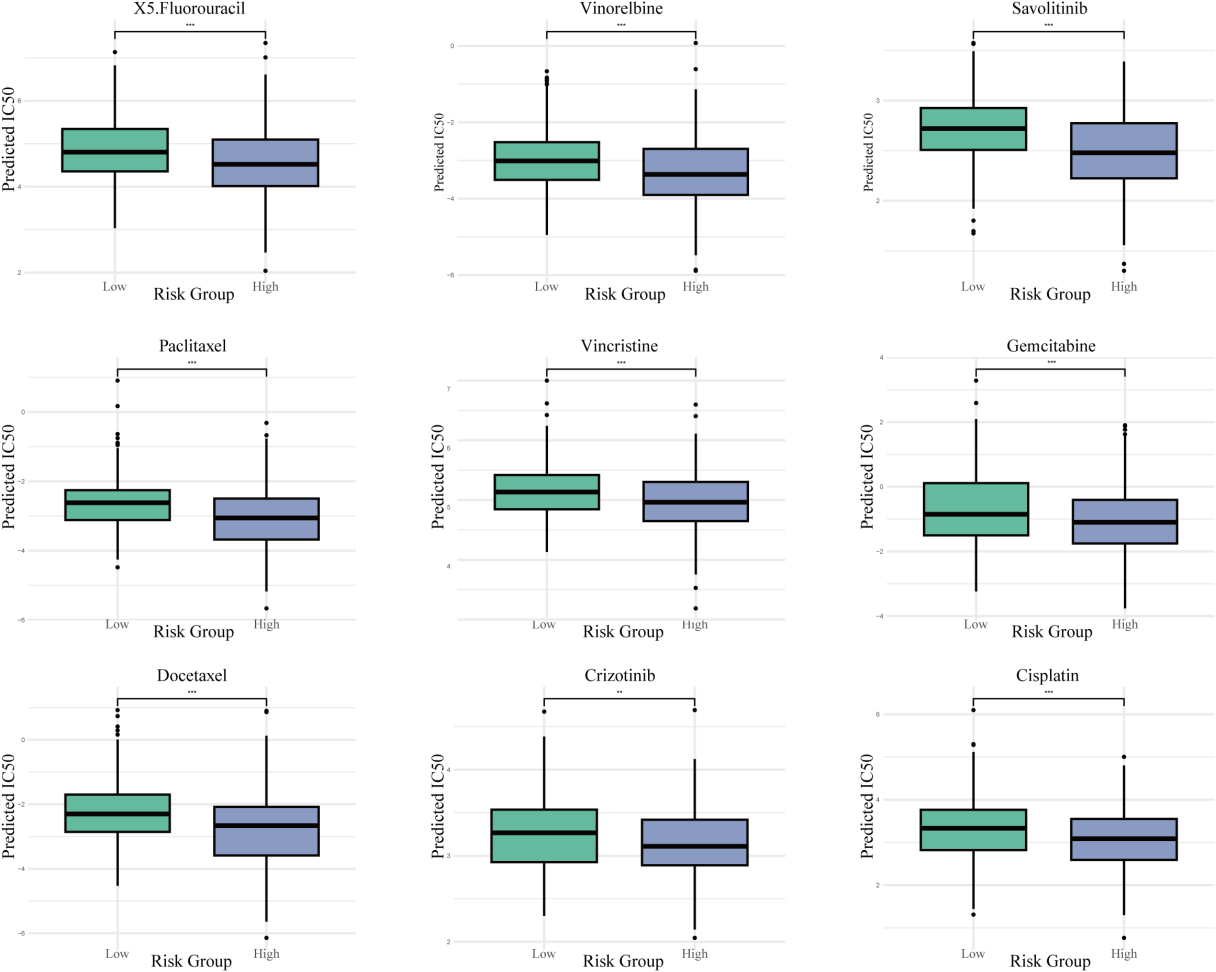
Projected drug sensitivity across risk categories. Boxplots contrasting the estimated half-maximal inhibitory concentration (IC50) values for a selection of chemotherapeutic and targeted agents between high- and low-risk populations. *** P < 0.001.

The prediction of drug responses utilizing the Genomics of Drug Sensitivity in Cancer database alongside the oncoPredict algorithm suggested that patients classified with elevated risk scores are more inclined to demonstrate increased sensitivity towards various chemotherapeutic and targeted treatment options. These results indicate that individuals within the high-risk cohort could potentially exhibit a more advantageous response to therapeutic agents such as Paclitaxel, Docetaxel, Vinorelbine, Cisplatin, Gemcitabine, Crizotinib, Savolitinib, Vincristine, and 5-Fluorouracil, as indicated by lower predicted IC50 values (**Figure 10**).

## 4 Discussion

This study first employed scRNA-seq to dissect the cellular composition of LUAD samples, identifying 11 major cell types and revealing that angiogenesis-related gene sets were significantly enriched in epithelial cells and macrophages. Through the novel combination of MR analysis and Cox regression modeling, ASPH and PTTG1 were pinpointed as core hub genes. These genes showed a positive genetic association with LUAD risk and were closely correlated with OS outcomes. To validate the biological relevance of these findings, ASPH and PTTG1 were confirmed to be significantly upregulated in LUAD through multi-level evidence, including scRNA-seq data, TCGA bulk RNA expression profiles, and protein-level validation by Western blotting. Based on their prognostic significance, a two-gene risk score model was constructed: Risk Score=0.151 × ASPH+0.236 × PTTG1, which demonstrated robust prognostic performance in both the TCGA dataset and two independent GEO cohorts. Furthermore, by analyzing TME immune characteristics, immune checkpoint expression, and chemotherapy drug IC50 data across different risk groups, we further investigated the immune landscape and potential therapeutic responses in LUAD patients with varying risk profiles. In summary, this study innovatively integrates bulk RNA-seq, scRNA-seq validation, and MR analysis, offering novel insights and potential clinical implications for prognostic assessment and therapeutic decision-making in LUAD.

ASPH is an α-ketoglutarate–dependent dioxygenase that promotes tumorigenesis through mechanisms such as enhancing angiogenesis, inhibiting apoptosis, and suppressing antitumor immunity (21–23). Elevated expression of ASPH has been observed in multiple malignancies, including non-small cell lung cancer, where its levels are significantly increased in bronchoalveolar lavage exosomes (24), as well as in pancreatic cancer, colorectal cancer, breast cancer, and hepatocellular carcinoma (25–28). Additionally, high ASPH expression has been linked to tumor recurrence, such as in retroperitoneal liposarcoma (RPLS), where it serves as an independent risk factor for recurrence (29), and has been identified as a potential target regulating tumor cell migration and invasion (30).

Mechanistically, ASPH enhances angiogenesis and metastasis by interacting with ADAM12/15, activating SRC kinase signaling, and facilitating MMP-mediated ECM degradation (31, 32). It also modifies the EGF-like repeats of the Notch receptor and its ligands, thereby activating the Notch signaling pathway (33). The ASPH–Notch axis promotes exosome secretion and facilitates the transfer of proteins associated with invasion, metastasis, metabolism, and immunosuppression (26, 28). Furthermore, ASPH inhibits GSK3β phosphorylation, interfering with upstream kinase communication, delaying cellular senescence, and promoting tumor progression (34).

These mechanisms are highly consistent with the features we observed in high-risk LUAD patients, including enhanced angiogenesis, reduced immune infiltration, high TMB, and suppressed immune checkpoint expression. Our study provides direct experimental evidence for these findings through *in vitro*​ functional assays. We demonstrated that specific knockdown of ASPH expression in two different LUAD cell lines significantly impaired their capacity for proliferation, migration, and invasion. This directly indicates that ASPH plays a critical, cell-autonomous role in maintaining the malignant phenotype of LUAD cells, thereby robustly complementing and validating the conclusions drawn from our multi-omics data and causal inference, and further solidifying the reliability of ASPH as a therapeutic target in LUAD. Currently, ASPH has emerged as a focal point in the research of several novel therapeutic targets. Inhibitory molecules targeting its enzymatic activity have been developed and have demonstrated anti-metastatic effects in preclinical models (22, 26, 35).

PTTG1, also known as human securin, is a multifunctional protein involved in angiogenesis, mitotic regulation, apoptosis, EMT, and MAPK signaling (36, 37). Overexpression of PTTG1 has been observed in multiple cancers—including pancreatic (38), prostate (39), LUAD (40), and hepatocellular carcinoma (41)—and is strongly associated with tumor progression and poor prognosis (42). Our study confirmed elevated PTTG1 expression in LUAD tumor tissues and its association with worse OS.

PTTG1 promotes angiogenesis by activating HIF-1α signaling, maintaining cancer stem cell (CSC) survival, and regulating vascular niche formation and metastasis (43, 44). It also upregulates angiogenesis-related proteins such as VEGF, p-PI3K/PI3K, p-eNOS/eNOS, and p-AKT/AKT, which are crucial for endothelial barrier remodeling (37, 45). As a β-catenin–interacting protein, PTTG1 stabilizes β-catenin and enhances its nuclear accumulation, leading to hyperactivation of the Wnt/β-catenin pathway (44), which plays a pivotal role in oncogenic transformation. In addition, PTTG1 can inhibit the TGF-β1/SMAD3 signaling pathway, thereby suppressing apoptosis and promoting tumor cell growth (46).

The study also revealed a complex and paradoxical immune phenotype in the high-risk group characterized. ESTIMATE analysis indicated lower immune scores but higher stromal scores in this group, while immune cell infiltration analysis showed significantly increased infiltration of activated memory CD4+ T cells, M0 macrophages, and resting natural killer cells. This suggests that the high risk group may harbor a microenvironment enriched with stromal components that support tumor growth, whereas the functionality of effector immune cells is likely suppressed or insufficiently activated. As a result, an immunosuppressive microenvironment emerges—one that appears immunologically active on the surface but is functionally impaired—facilitating tumor immune evasion. This observation is consistent with findings by Hong et al. (47). Such a “cold tumor” microenvironment is typically unresponsive to immune surveillance, allowing tumors to escape recognition and elimination by the immune system (48). Clinically, immune “hot tumors” typically exhibit higher immune activity, lower disease stages, and better survival outcomes compared to “cold tumors” (49).

Notably, tumors in the high risk group exhibit elevated CYT and TMB, yet show downregulation of several key immune checkpoint genes, such as CTLA4, CD47, BTLA, and ICOS. This paradoxical phenotype of high TMB coexisting with low immune infiltration suggests a sophisticated immune escape mechanism. Tumors in the high-risk group, despite possessing a high number of neoantigens that should trigger an immune response, appear to have established an immunosuppressive microenvironment. This aligns perfectly with the known functions of ASPH and PTTG1. Both genes are implicated in promoting aberrant tumor angiogenesis, which creates a physical barrier and a hypoxic, acidic milieu that hinders T-cell infiltration and function. Furthermore, they can drive the secretion of immunosuppressive cytokines and recruit regulatory immune cells, thereby actively dampening anti-tumor immunity and uncoupling mutational load from effective immune surveillance (44, 50). Therefore, conventional immune monotherapy may have limited efficacy in high-risk patients, and combination strategies involving anti-angiogenic therapies should be considered to remodel the tumor immune microenvironment and enhance the effectiveness of immunotherapy (5). This hypothesis is consistent with the emerging trend of combining anti-angiogenic agents with immunotherapy in LUAD treatment.

Our study, in conjunction with previous literature, reveals that high risk patients are not necessarily more resistant to all treatments; on the contrary, they may exhibit increased sensitivity to certain therapies. Drug sensitivity predictions suggest that the high risk group may be more responsive to various chemotherapeutic agents (such as cisplatin, gemcitabine, and 5-fluorouracil) as well as targeted therapies (including crizotinib and savolitinib). GSEA results indicate enrichment of pathways related to cell cycle regulation, DNA replication, and hypoxic response in the high risk group, suggesting that tumor cells in this group are highly proliferative and thus more vulnerable to DNA-targeting agents. For example, cisplatin induces apoptosis by forming DNA crosslinks at purine bases and disrupting DNA repair mechanism (51),which may explain the higher sensitivity of the high-risk group to this drug. Moreover, ASPH and PTTG1 may themselves play regulatory roles in drug response pathways, thereby influencing tumor cell sensitivity to specific treatments. These findings provide a potential rationale for precision therapy in high-risk patients, and future studies integrating drug sensitivity databases, experimental data, and functional assays are warranted to further validate the relationship between these mechanisms and treatment responses.

This study is the first to validate, at the protein level, the significant overexpression of ASPH and PTTG1 in LUAD tumor tissues, which is consistent with previous findings based on single-cell transcriptomics and bioinformatic analyses. This experimental confirmation enhances the reliability of our conclusions and suggests that ASPH and PTTG1 may play oncogenic roles in LUAD pathogenesis. Future research should expand the sample size and incorporate longitudinal clinical follow-up to further evaluate the potential of these markers in LUAD diagnosis, prognosis, and personalized therapeutic decision-making.

One of the key strengths of this study lies in the integration of MR with multi-omics data to identify potential therapeutic targets and elucidate underlying mechanisms, particularly in exploring causal relationships between genes and LUAD. This approach offers a clear advantage over traditional studies that rely on single data sources. However, several limitations remain to be addressed in future research. First, while our *in vitro* assays provide initial functional support, the study lacks *in vivo* validation using animal models, which is essential to confirm the roles of ASPH and PTTG1 in a physiological tumor microenvironment. Second, although we performed rigorous batch effect correction and external cohort validation, the integration of datasets from different platforms and cohorts may still introduce batch effects and heterogeneity, potentially affecting the robustness of our findings. Lastly, while MR supports a causal relationship between ASPH/PTTG1 and LUAD phenotypes, the underlying genetic data are largely derived from European populations, which may limit the generalizability of the results. Moreover, the use of whole-blood eQTL data from the eQTLGen Consortium, rather than lung tissue-specific data, may not fully capture the tissue-specific regulatory patterns of gene expression, which could influence the causal estimates. Future studies should focus on validating the functional roles of ASPH and PTTG1 in angiogenesis and immune evasion through *in vitro* and *in vivo* experiments and exploring their potential as combined immunotherapy targets. Specifically, the absence of direct experimental validation for PTTG1’s function is a significant gap that we plan to address in subsequent studies. Similarly, while our multi-omics analysis strongly implicates these genes in angiogenesis, direct phenotypic validation through assays such as tube formation was not performed and remains an important area for future investigation.

## 5 Conclusion

ASPH and PTTG1 not only play critical roles in angiogenesis and immune regulation in LUAD, but also demonstrate strong prognostic predictive capabilities. This study provides a potential biological foundation and therapeutic targets for personalized treatment of LUAD, particularly offering promising avenues for the development of combined immunotherapy and anti-angiogenic strategies.

## Data Availability

Publicly available datasets were analyzed in this study. This data can be found here: The datasets employed in the present study are openly accessible to the public. The bulk RNA-seq data pertaining to LUAD were acquired from TCGA through the Genomic Data Commons portal (https://portal.gdc.cancer.gov/) as well as from the GEO using accession numbers GSE37745, GSE41271, and GSE131907. GWAS summary statistics were obtained from the FinnGen database (https://www.finngen.fi/en), and eQTL data were sourced from the eQTLGen Consortium (https://www.eqtlgen.org). The analysis code supporting the findings of this study is accessible at: https://github.com/KaiyangCH1/kaiyangch1.git.
